# Diagnosis, management and outcome of Spinal Cord Injury without Radiographic Abnormalities (SCIWORA) in adult patients with trauma: a case series

**DOI:** 10.5339/qmj.2021.67

**Published:** 2021-11-23

**Authors:** Suhail Yaqoob Hakim, Lubna Gamal Altawil, Ahmed Faidh Ramzee, Mohammad Asim, Khalid Ahmed, Motasem Awwad, Ahmed El-Faramawy, Monira Mollazehi, Ayman El-Menyar, Mohamed Ellabib, Hassan Al-Thani

**Affiliations:** ^1^Department of Surgery, Trauma Surgery Section, Hamad General Hospital (HGH), Doha, Qatar E-mail: aymanco65@yahoo.com; ^2^Department of Emergency Medicine, HGH, Doha, Qatar; ^3^Department of Surgery, Trauma surgery Section, Clinical research, HGH, Doha, Qatar; ^4^Department of Surgery, Trauma Surgery Section, Qatar Trauma registry, HGH, Doha, Qatar; ^5^Clinical Medicine, Weill Cornell Medical College, Doha, Qatar; ^6^Department of Surgery, Acute Care Surgery, HGH, Doha, Qatar

**Keywords:** Spinal cord injury without radiographic abnormality, magnetic resonance imaging, Computed Tomography, trauma, management, outcomes

## Abstract

Background: Spinal cord injury without radiographic abnormality (SCIWORA) in adults causes diagnostic and prognostic dilemma as radiography and/or computed tomography does not clearly detect bone lesions during the initial assessment. Herein, we report our experience on 11 spinal cord injury cases without radiographic abnormality, regarding the clinicoradiological features, management, and outcomes.

Methods: We conducted a case series of adult patients with SCIWORA who were admitted at the level 1 trauma center at Hamad General Hospital from January 2008 to July 2018. All patients underwent initial head and spine X-ray imaging, computed tomography, magnetic resonance imaging, and 12 months of clinical follow-up.

Results: Eleven patients (mean age, 46.5 ± 14.4 years) met the criteria of SCIWORA. The neurologic status on admission and 12 months after hospital discharge were classified according to the American Spinal Injury Association (ASIA) impairment scale (AIS). On admission, 6 (54.5%) patients had ASIA grade C: 2 (18.2%) each had AIS grade D and B and 1 (9.1%) had AIS grade A. Five cases were treated conservatively with rehabilitation and physiotherapy, and five were treated surgically by anterior cervical discectomy with fusion. One patient who declined surgery was managed with a sternal occipital mandibular immobilizer brace and underwent rehabilitation.

Conclusion: SCIWORA requires higher clinical suspicion and thorough neurological and radiologic assessment to prevent secondary spinal cord injuries and complications.

## Introduction

Traumatic spinal cord injury (TSCI) is associated with higher morbidity, affecting young individuals causing severe and permanent disabilities that incur huge healthcare burden on the patients.^1–3^ TSCI is a clinicoradiological diagnosis of post-traumatic neurological weakness with bony deformity based on radiography and computed tomography (CT) and confirmed by magnetic resonance imaging (MRI).^3^ Spinal cord injury without radiographic abnormality (SCIWORA) is a recognized form of SCI, infrequently reported in adults and characterized by the absence of any radiographically evident fracture or dislocation.^4^ It is defined as spinal cord lesions that are observed by MRI but do not show lesions in plain radiography and/or CT.^4^ This can lead to a clinical dilemma and may lead to serious clinical complications. It is more frequently reported among children^5^ than in adults, which commonly involves injury to the cervical spine, followed by the thoracolumbar spine.^6^ The reported incidence of SCIWORA among adult SCI cases ranges from 10% to 12%,^7^ and falls from height, motor vehicle accidents, and sports-related injuries are the major injury mechanisms.^8^ The epidemiology and pathophysiology of SCIWORA differ among adults and children, with most adult patients presented with radiographic abnormalities showing degenerative changes.^9^ The diagnosis depends on the rapid and progressive onset of neurological symptoms and MRI findings. Careful physical examination on follow-up assessment might help in identifying the neurological deficits, which may appear few days post-trauma.^6^ Therefore, it is important for treating physicians to be aware of this clinical entity to help them in determining appropriate diagnosis and making decisions on the management of such infrequent cases. Herein, we report a case series of 11 adult SCIWORA cases from our institution and describe the clinicoradiological features, management, and outcomes at a level 1 trauma center.

## Methods

We conducted a case series of all admitted adults patients with SCIWORA at the level 1 trauma center of Hamad General Hospital in Qatar from January 2008 to July 2018. The Medical Research Center (MRC-04-20-321) at Hamad Medical Corporation approved this study with a waiver of informed consent, as there was no direct contact with patients and data were anonymously collected. All study patients underwent initial spine X-ray imaging, CT scan, magnetic resonance imaging, and 12 months of clinical follow-up. The neurologic status on admission and 12 months after hospital discharge was classified according to the American Spinal Injury Association (ASIA) impairment scale. The inclusion criteria were as follows: patients with acute trauma admitted to the emergency department with spine injuries (Glasgow Coma Scale [GCS] of 15), presented with neurological deficit plus normal spine X-ray imaging/CT but abnormal MRI on initial presentation. Exclusion criteria included patients who died prior to the initial CT or cases without images for review. Patients aged < 18 years, pregnant women, those with positive CT findings, and GCS < 15 were also excluded.

We used the classification described by Sharma et al. to categorize neural injuries.^10^ On tbl2-weighted (T2W) images, pattern I (hematoma) is defined as a large central area of hypo-intensity surrounded by a thin rim of hyperintensity, pattern II (edema) is characterized by an area of hyperintensity, and pattern III (cord contusion) is characterized by a thin area of central hypo-intensity and a thick rim of hyperintensity.

The variables of interest were patient demographic characteristics (age and gender), mechanism of injury, ASIA impairment scale, CT and MRI findings, treatment, and 12-month clinical evaluation.

Data were presented as proportions and mean ( ± standard deviation; SD) as appropriate. Data analysis was performed using the Statistical Package for Social Sciences version 21 (IBM Corp., Armonk, NY, USA).

## Results

Demographic data, mechanism of injury, clinicoradiological characteristics, and neurological status on hospital discharge of patients with adult SCIWORA are summarized in Tables 1 and 2. The majority of the patients were adults, predominantly males (10 of 11) with a mean age of 46.5 ± 14.4 years. The most common mechanism of injury was a fall from a height, followed by fall of a heavy object and motor vehicle crash (MVCs). The ASIA impairment scale (AIS) was used to classify the neurologic state on admission and 12 months after hospital discharge. On admission, 6 (54.5%) patients had ASIA grade C, 2 (18.2%) each had AIS grade D and B, and 1 (9.1%) had AIS grade A. Only 2 (18.2%) patients who had isolated contusion without any edema, hematoma, and disc or ligamentous pathology on MRI showed complete recovery.

**Figure 1.**
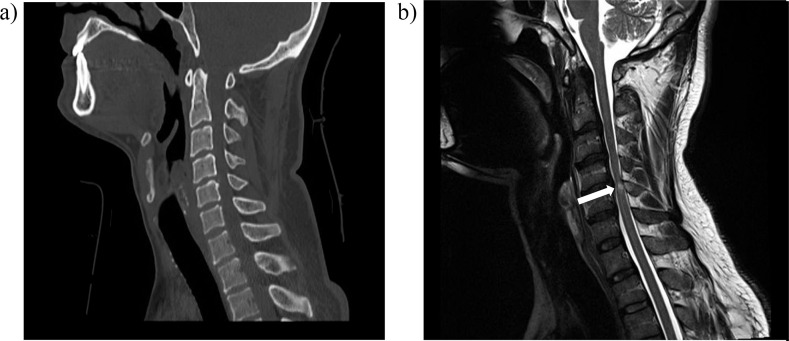
A case of SCIWORA. **(a)** Normal computed tomography of the cervical spine. **(b)** tbl1- and tbl2-weighted magnetic resonance imaging of the cervical spine (same patient) with spinal cord contusion (white arrow)

In comparison, 9 (81.8%) patients showed either a partial recovery or no recovery at all. Surgical intervention as anterior cervical discectomy and fusion (ACDF) was performed in 5 of 11 (45.5%) patients who had intervertebral disc involvement bulge or prolapse besides cord injury. All patients with cervical spine trauma with neurological deficit were admitted to the trauma intensive care unit for initial close observation. Figure 1 shows an example of a case of SCIWORA.

## Discussion

SCIWORA in adults is an uncommon condition that more frequently occur in the pediatric population, mainly affecting the cervical and thoracolumbar spines.^11^ The increased frequency of SCIWORA in the pediatric age group with a predilection of SCI is attributed to greater cervical mobility, higher head-to-body ratio, and ligamentous laxity.^7^

In adults, SCIWORA has clinical relevance because of age-related degenerative changes in the cervical spine that may play a role in etiopathogenesis in addition to traumatic hyperextension or hyperflexion of the spinal cord.^12^ In our series, 10 of 11 patients were male (9 were <  50 years old). Similarly, a study reported that 10 of 12 adult patients with SCIWORA were men aged 22–58 years.^10^ An earlier systematic review of SCIWORA cases suggested male (68.5%) predominance in comparison to female gender (31.5%).^13^ In the present case series, all patients with SCIWORA sustained lower cervical spine injuries. Consistent with our findings, an earlier study by Sharma et al.^10^ reported involvement of cervical spine injuries among all 12 adult SCIWORA cases. Notably, thoracic (9.5%) or lumbar (1.5%) spine injuries are less commonly identified.^7^ However, case reports of SCIWORA also rarely diagnosed thoracolumbar spine injuries in the adult population.^14^

Vertebral fractures are mostly associated with high-impact trauma or to spinal rigidity in adults causing structural instability. In particular, common mechanisms of SCIWORA include blunt trauma such as MVC, falls from a height, sports injuries, and violence.^6,15^ This is also evident from our series with five cases of work-related falls from a height, four had a heavy object fall onto them, and two sustained injuries because of MVC.

The current literature suggests age-based predilection of injury mechanism, i.e., sports injuries and MVC are more common in the pediatric group,^16^ and falls from a height are predominant among adult SCIWORA cases.^17^ Another case series of adult SCIWORA cases reported MVC (n = 5) and fall from a height (n = 4) as frequent injury mechanisms.^10^

Thorough clinical evaluation and neurological deficit assessment may guide emergency physicians to investigate spinal injuries further.^18^ Compared with earlier case reports, the diagnostic rate of SCIWORA among adults with neurological deficit has increased with the advent of MRI, which has superior visualization of pathomorphological soft tissue changes.^19^ Notably, CT can identify bony pathologies of the vertebrae, which are not always evident from the conventional X-ray imaging. However, suspected cases with SCIWORA should undergo MRI, which is the modality of choice for directly evaluating SCI characterized by pathomorphological soft tissues. These changes could not be found on radiological diagnostic (X-ray or CT images) despite neurological deficits.^6^

Neurological dysfunction after anatomical deformities secondary to compression and hyperextension of the spinal cord is referred to as acute spinal cord trauma.^20^ Abnormalities detectable in MRI could be intraneural such as edema, hemorrhage, and contusion, and extraneural such as disc herniation, bulging of the ligamentum flavum, and soft tissue swelling of the prevertebral region.^21^ In our series, intraneural abnormalities on MRI findings were contusion in 6 (54.5%) patients, edema in 5 (45.4%) patients, and hematoma in 4 (36.4%) patients. In addition, frequent extraneural abnormalities were disc herniation (n = 7) and ligament injury (n = 2).

Therefore, a post-injury neurological examination is useful for assessing the level of SCI to monitor the prognosis of the patients.^22^ The ASIA is the commonly used tool to grade neurological impairment in patients with SCIWORA.^23^ An earlier retrospective study of 48 adult patients with SCIWORA showed ASIA grade D as the most frequent neurological deficit on admission, followed by grades C and grade B.^24^ On a 1-year follow-up post-injury, the authors reported complete recovery in neurological status of patients who had ASIA grades C and D on initial admission. In our series, six patients had ASIA grade C, two had ASIA grades D and B, and only one patient had ASIA grade A. Upon hospital discharge, only two patients attained complete recovery (ASIA grade E) who initially presented with ASIA grade D on admission. Similar to our observation, Sharma et al.^10^ reported ASIA C in seven patients, followed by ASIA B in three cases. Our findings indicate that patients with less severe SCI are more likely to achieve complete recovery. By contrast, patients with severe injuries had residual or persistent neurological dysfunction, which is in agreement with a previous study.^6^

Neck immobilization and steroid therapy are primary conservative approaches to treat SCIWORA cases. Moreover, surgery can be considered in selected cases with clear indication of extraneural abnormalities, such as ligamentous injury and/or cord compression that necessitates operative intervention.^6,7^ In our series, six patients underwent nonsurgical treatment and five patients were treated surgically with ACDF to remove a herniated or degenerative disc of the spinal cord. However, none of our patients received steroids.

This study was limited by its retrospective design and relatively small sample size. Therefore, further prospective multicenter studies are needed.

## Conclusion

A definite diagnosis of SCIWORA in adults necessitates higher clinical suspicion and thorough neurological assessment together with MRI findings, as delayed diagnosis may lead to poor neurological outcomes. This clinical entity is a diagnostic and prognostic dilemma for emergency physicians, so its awareness is crucial to prevent secondary SCIs and associated crippling complications. The initial neurological deficit and magnitude of SCI on MRI are suggestive of the prognosis. Therefore, patients with SCIWORA with less severe injuries are likely to achieve complete recovery. We recommend early MRI for definitive diagnosis and prompt neuroprotective measures to prevent secondary SCI that may cause further neurological deterioration for a better prognosis. On initial presentation, most patients with incomplete neurological deficit (ASIA A–C) had residual weakness that needs longer follow-up to expect a complete recovery.

### Acknowledgments

We thank the registry database team in the Trauma Surgery Section, Hamad General Hospital, Qatar.

### Funding

None

### Conflict of interest

None

### Informed consent

As data were collected retrospectively and anonymously without direct contact with patients, a waiver of consent was granted.

### Data availability

All data were given in the results and tables.

### Authors’ contributions

All authors contribute substantially in the study design, data interpretation, manuscript drafting, and approval.

## Figures and Tables

**Table 1:** Demographics, mechanism of injury, and clinical presentation

Patient	Age (years)	Gender	Mechanism of injury	Clinical presentation

1	35	Male	Fall of heavy object	Bilateral upper limb weakness

2	36	Male	Motor vehicle crash	Numbness on the right side of the body involving both upper and lower limbs.

3	36	Male	Fall of heavy object	Numbness in both upper limbs and inability to void urine

4	37	Male	Fall of heavy object	Upper limb weakness and inability to walk

5	38	Male	Fall of heavy object	Unable to move all limbs and to pass urine

6	42	Male	Fall from height	Unable to move all limbs and had no sensations

7	42	Male	Fall from height	Numbness in his hands and weakness in both lower limbs

8	45	Female	Motor vehicle crash	Numbness and weakness of right upper limb

9	49	Male	Fall from height	Bilateral upper and lower limb weakness

10	72	Male	Fall from standing height	Bilateral upper and lower limb weakness

11	79	Male	Fall from the stairs at home	Bilateral upper limb weakness


**Table 2:** Clinicoradiological characteristics and treatment of patients with SCIWORA

Patient	ASIA grade on admission	Segment of spinal injury	Contusion	Edema	Hematoma	Disc herniation	Bone/ ligament injury	Treatment	Neuro deficit /ASIA grade at 12 months

1	D	C3–C4	Yes	No	No	No	No/ No	Nonoperative	Recovered /E

2	D	C4–7	Yes	No	No	No	No/ No	Nonoperative	Recovered /E

3	C	C4–C6	No	Yes	Yes	Yes	No/ No	ACDF	Residual weakness/D

4	C	C5	Yes	Yes	No	Yes	No/ No	ACDF	Residual weakness/D

5	B	C6	No	No	Yes	Yes	No/ No	ACDF	Residual weakness/ C

6	A	C3–C4	No	Yes	No	Yes	No/Yes	SOMI Brace (refused Surgery)	Residual weakness/ B

7	C	C4–C5	No	Yes	No	Yes	No/No	ACDF	Residual weakness/ D

8	C	C3–5	Yes	No	No	Yes	No/ No	ACDF	Persistent weakness/ C

9	C	C3–C4	Yes	No	No	No	No/ No	Nonoperative	Persistent weakness/ C

10	B	C2–C6	Yes	No	Yes	No	No/ No	Nonoperative	Residual weakness/ C

11	C	C5–C7	No	Yes	Yes	Yes	No/Yes	Nonoperative	Persistent weakness/ C


ASIA, American Spinal Injury Association Impairment Scale; ACDF, anterior cervical discectomy and fusion; SOMI, sternal occipital mandibular immobilizer

